# Design and Simulation of Suspension Leveling System for Small Agricultural Machinery in Hilly and Mountainous Areas

**DOI:** 10.3390/s25247447

**Published:** 2025-12-07

**Authors:** Peng Huang, Qiang Luo, Quan Liu, Yao Peng, Shijie Zheng, Jiukun Liu

**Affiliations:** College of Mechanical Engineering, Chongqing Three Gorges University, Chongqing 404100, China; hpeng47@163.com (P.H.); 18875566368@163.com (Q.L.); boometro@163.com (Y.P.); 19115182845@163.com (S.Z.); liujiukun9123@163.com (J.L.)

**Keywords:** hilly and mountainous, agricultural machinery, active levelling, fuzzy PID, dynamic simulation

## Abstract

To address issues such as chassis attitude deviation, reduced operational efficiency, and diminished precision when agricultural machinery operates in complex terrains—including steep slopes and fragmented plots in hilly and mountainous regions—a servo electric cylinder-based active suspension levelling system has been designed. Real-time dynamic control is achieved through a fuzzy PID algorithm. Firstly, the suspension’s mechanical structure and key parameters were determined, employing a ‘servo electric cylinder-spring-shock absorber series’ configuration to achieve load support and passive vibration damping. Secondly, a kinematic and dynamic model of the quarter-link suspension was established. Finally, Simulink simulations were conducted to model the agricultural machinery traversing mountainous, uneven terrain at segmented stable operating speeds, thereby validating the suspension’s control performance. Simulation results demonstrate that the system maintains chassis height error within ±0.05 m, chassis height change rate within ±0.2 m/s, and response time ≤ 0.8 s. It rapidly and effectively counteracts terrain disturbances, achieving precise chassis height control. This provides theoretical support for designing whole-vehicle levelling systems for small agricultural machinery in hilly and mountainous terrains.

## 1. Introduction

The hilly and mountainous regions of China account for approximately 35% of the nation’s total arable land area and 34% of its sown acreage, serving as a vital production zone for crops such as soybean and maize. However, due to terrain constraints—including fragmented, irregularly shaped plots, numerous slopes, complex topography, and challenging road transport conditions—the overall mechanisation rate in these areas remains below 50% [1,2,3,4]. Additionally, flat cultivated land with gradients of 2° or less and gently sloping land between 2° and 6° present minimal cultivation challenges, allowing standard tractors to perform stable and efficient operations. Hilly terrain between 6° and 15° is better suited for small to medium-sized agricultural machinery. Slopes of 15–25° require exclusively small-scale machinery, with attitude levelling systems becoming indispensable. Consequently, addressing mechanisation for 6–15° hilly terrain and 15–25° slopes—specifically small-scale mechanisation for fragmented plots in hilly regions—is an urgent priority [5]. To enhance the operational stability of agricultural machinery in complex environments such as hilly and mountainous terrains, meet the precision requirements for sowing crops like soybean and maize [3,4], and improve both the quality and efficiency of agricultural operations, innovative development of levelling chassis technology for small-scale agricultural machinery is feasible. This technology adjusts the chassis’ tilt angle to maintain chassis stability on uneven slopes, ensuring continuity and precision in agricultural operations [5].

Research into agricultural machinery levelling systems has achieved significant progress at present. Gao Moyao et al. [6] developed a self-propelled universal chassis suitable for agricultural operations in hilly and mountainous regions. This chassis employs hydraulic differential height technology, utilising tilt sensors to determine the vehicle’s inclination state. By controlling the stroke of hydraulic cylinders at the front and rear, it alters the configuration of the parallelogram linkage mechanism to achieve vehicle levelling. Yang Fuzeng’s team [7,8] enhanced the walking system of tracked tractors, enabling vertical counter-adjustment of both tracks according to terrain. Through lateral attitude adjustment, the tractor maintains body level and performs contour line operations on slopes ranging from 0° to 23° [9,10]. Building upon prior research, a parallelogram-based attitude adjustment mechanism was designed, undergoing simulation validation and physical model testing [11]. Han Zhenhao et al. [12] designed an adaptive mountain orchard transport vehicle capable of real-time automatic adjustment of the machine’s centre of gravity to achieve equilibrium based on terrain conditions. This design prevents cargo loss due to road undulations, enhancing transport efficiency and speed while delivering superior slope performance and terrain adaptability. Ning Pucai [13] developed a longitudinal centre-of-gravity adjustment mechanism for electric tractors, shifting the front battery pack’s position to alter the tractor’s longitudinal centre of gravity, thereby improving traction efficiency and stability during uphill/downhill operations. John Deere Company of the United States [14] designed a six-wheel harvester employing an articulated steering structure. The front and rear frames are connected via composite hinge technology for steering, enhancing chassis flexibility. Suspension oscillation improves wheel-to-road contact. The MACH 4R mountain tractor produced by Italy’s Antonio Carolla [15] features articulated and articulated-twisting capabilities. It delivers robust power and adapts to varied uneven terrain, demonstrating good performance on gentle slopes. However, this model’s slightly larger frame and lack of overall levelling capability compromise safety and mountain adaptability when operating on steep gradients. The Sky-Jump-V950 mountain tractor produced by Italy’s BCS [15] incorporates a waist-bending mechanism. This structure enables a turning angle of 70° and provides excellent traction and stability during operations on steep slopes. Bałchanowski et al. [16,17] investigated a hybrid levelling system combining efficient wheeled movement on flat terrain with the ability to traverse obstacles by walking. This enables wheel-legged robots to automatically level their chassis when traversing uneven surfaces, effectively maintaining a constant height relative to the ground. Significant research progress has also been made in control algorithms for chassis levelling systems. Peng He et al. [18,19] designed a self-levelling system for the body of a hilly terrain tractor. Based on the tractor and its four-point body levelling mechanism, the levelling system employs hydraulic cylinders driven by a fuzzy PID control algorithm for real-time dynamic levelling. To ensure safe operation of agricultural machinery in hilly terrain while enhancing work efficiency and ride comfort, Qi Wenchao et al. [20,21] proposed an automatic levelling mechanism and control system for hilly terrain tractors based on a dual-closed-loop fuzzy PID control algorithm. In summary, significant progress has been made in designing and optimising automatic levelling systems for agricultural machinery in hilly terrain. However, practical applications still face certain limitations. A comparative table of levelling techniques for agricultural machinery in hilly and mountainous terrains is presented in Table 1.

Current levelling principles primarily include hydraulic differential height type [6,7,8,9,10], parallel four-bar linkage [6,11], adjustable centre of gravity mechanism [12,13], bending and twisting the waist [14,15], and omnidirectional levelling [16,17,18,19]. During operations in hilly and mountainous terrains, the vehicle posture of agricultural machinery undergoes arbitrary changes, necessitating both lateral and longitudinal levelling. While hydraulic differential height type and parallel four-bar linkage achieve lateral levelling, they cannot accommodate longitudinal levelling, resulting in poor performance when encountering complex slopes involving both roll and pitch. The adjustable centre of gravity mechanism necessitates additional guide rail design, occupies space beneath the machinery, and increases overall height. The bending and twisting the waist can only passively adapt to terrain changes and cannot achieve active levelling. Omnidirectional levelling mechanisms, utilising either four-wheel independent hydraulic levelling or multi-level chassis hydraulic systems, can simultaneously correct roll angles and pitch angles, achieving full-posture horizontal alignment of the vehicle body.

Currently, some studies have employed electric cylinders as actuators for agricultural machinery levelling systems, laying the groundwork for technological advancement in this field. Jia Xinle et al. [22] proposed an automatic levelling system for small and medium-sized agricultural machinery in hilly and mountainous regions. They designed a three-point levelling mechanism employing servo electric cylinders as actuators to achieve automatic adjustment of the working chassis’ tilt angle. Static and dynamic testing validated the system’s feasibility for automatic levelling applications. Zhang Fugui et al. [23] designed an automatic levelling control system for the levelling mechanism of a tracked tractor. Angle data collected via an inclination sensor adjusted motor rotation and servo electric cylinder extension/retraction to achieve automatic levelling of the vehicle platform. However, inconsistent lateral and longitudinal levelling structures resulted in prolonged levelling times and significant overshoot. Yang Huabing et al. [24] enhanced the tractor’s levelling structure by designing a fuzzy PID controller based on X-axis and Y-axis speed levelling control of the tractor body. This regulates the extension and retraction of the body levelling servo electric cylinder, achieving reduced system oscillations and shorter levelling times. Ding Renkai et al. [25] devised an omnidirectional adjustment system for a ‘double-layer frame’ tracked agricultural chassis. Employing adaptive sliding control with servo electric cylinders as drive components, this system enables large-angle levelling control in steep slope operating environments.

In summary, a levelling system suitable for small agricultural machinery can be designed using omnidirectional levelling based on servo electric cylinders, capable of addressing complex operating conditions in hilly and mountainous terrains.

This paper addresses small agricultural machinery for hilly and mountainous terrains, focusing on quarter-lift suspension systems. It designs an active levelling suspension driven by servo electric cylinders, establishes suspension dynamics and kinematic models, and employs a fuzzy PID control algorithm for chassis height regulation. Simulation verifies the quarter-lift levelling system’s adaptability to hilly and mountainous operational environments.

## 2. Levelling Control System and Key Executing Agencies

### 2.1. Levelling Control System

**Figure 1:**
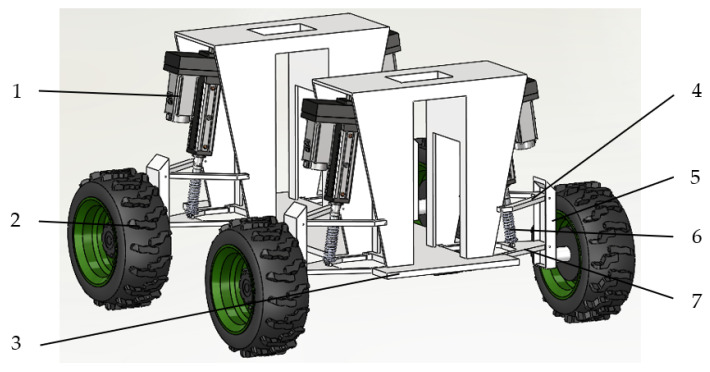
Schematic diagram of small agricultural machinery chassis for hilly and mountainous terrains. 1. Reciprocating servo electric cylinder 2. Wheel 3. Chassis 4. Upper axle beam 5. Connecting member 6. Shock absorber spring 7. Lower axle beam.

**Figure 2:**
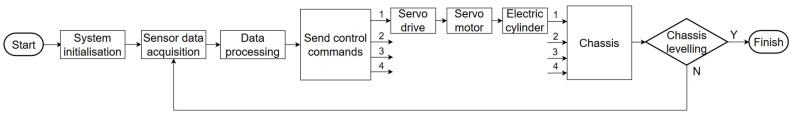
Schematic diagram of the levelling control system.

The schematic diagram of the small agricultural machinery chassis for hilly terrain is shown in Figure 1. A dual-axis inclinometer sensor is mounted at the geometric centre of the chassis, transmitting the platform’s X-axis and Y-axis angles to the Microcontroller Unit (MCU) via CAN bus. The servo motor incorporates a rotary encoder, enabling real-time acquisition of the electric cylinder rod position, which is then transmitted to the MCU. The control system processes the feedback signals and applies corresponding control algorithms to the electric cylinder. This regulates the cylinder rod’s extension and retraction to adjust the vertical displacement of the suspension, thereby achieving chassis levelling. The operational principle of the levelling control system is illustrated in Figure 2.

### 2.2. Key Executing Agencies

Hydraulic systems are well suited for agricultural machinery applications involving heavy loads due to their capacity to deliver substantial thrust. Furthermore, their control methods are well established, allowing straightforward regulation of cylinder rod extension and retraction by adjusting hydraulic fluid flow and pressure. Consequently, hydraulic systems are predominantly employed in the design of agricultural machinery levelling systems. However, the complex terrain of hilly and mountainous regions imposes limitations on the application of hydraulic systems, whereas electric cylinders do not require the intricate components such as pumps, valves, and hydraulic piping found in hydraulic systems. The distinctions between hydraulic cylinders and electric cylinders are outlined in Table 2.

Compared to hydraulic cylinders, electric cylinders possess distinctive performance characteristics:

(1) High transmission efficiency: Electric cylinders employing precision ball screws or planetary roller screws eliminate numerous complex mechanical structures, significantly enhancing transmission efficiency to exceed 90%. Conversely, hydraulic cylinders feature multiple energy conversion stages, with combined effects of fluid compression, flow resistance, leakage, and mechanical friction resulting in lower transmission efficiency, typically below 50% [26].
(2) Rapid response speed: Electric cylinders offer adjustable linear operating speeds across a broad range, with stable low-speed performance. Hydraulic cylinders typically operate at speeds up to 35 mm/s, whereas standard electric cylinders can reach 55 mm/s. Models employing planetary roller screws achieve speeds as high as 2000 mm/s [26], demonstrating a pronounced velocity advantage.
(3) High positioning accuracy: Servo electric cylinders achieve precise positioning of approximately 0.01 mm through servo control, demonstrating exceptional positioning accuracy [26]. Electric cylinders attain high positioning accuracy even in semi-closed-loop operation. Conversely, hydraulic cylinders, affected by factors such as fluid compressibility, leakage, temperature variations, and mechanical friction, require closed-loop control systems to achieve comparable positioning precision.
(4) Simple structure, compact footprint, and convenient maintenance [27]: Electric cylinders primarily comprise a motor and a nut-screw mechanism, featuring straightforward construction and compact dimensions that minimise workspace requirements. Their simplicity facilitates rapid fault diagnosis during malfunctions and simplifies routine maintenance. In contrast, hydraulic cylinders necessitate periodic management of fluid contamination, replacement of hydraulic oil and seals, inspection for pipeline leaks and component wear, and rely on specialised operation to ensure system stability.
(5) High reliability and safety [28]: Electric cylinders can incorporate advanced sensor systems and various stroke control devices to monitor and provide feedback on operational status, preventing accidents.
(6) Stable operation and extended service life: Electric cylinders utilising ball screws or planetary roller screws significantly reduce friction in the transmission components, enhancing operational stability and prolonging service life.
(7) Exceptional environmental adaptability: Electric cylinders exhibit minimal sensitivity to ambient temperatures, functioning reliably in extreme conditions including low/high temperatures, rain, and snow. In contrast, hydraulic cylinders are significantly affected by temperature variations, which alter hydraulic fluid viscosity, accelerate seal wear and leakage, and may compromise fluid compressibility, thereby disrupting pressure transmission and motion control precision.

In summary, electric cylinders offer a range of advantages including high transmission efficiency, strong adaptability, high precision, simple structure, rapid response, and stable operation. They can also deliver substantial torque output, making them well suited to replace hydraulic cylinders as suspension actuators. Consequently, servo electric cylinders are selected as the key actuators for the levelling systems of small agricultural machinery in hilly and mountainous terrains.

**Figure 3:**
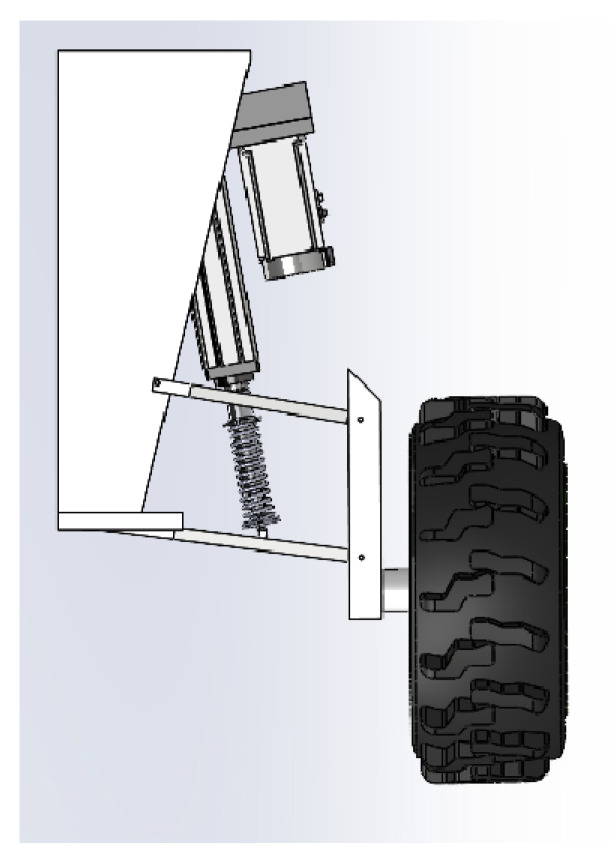
Schematic diagram of quarter suspension.

The quarter-suspension structure, as illustrated in Figure 3, comprises a servo electric cylinder, springs, shock absorbers, wheel assemblies, and connecting brackets. The servo electric cylinder serves as the primary actuator, responsible for vertical suspension extension and retraction to regulate chassis height, with real-time extension feedback provided via a rotary encoder. The spring and shock absorber are connected in parallel before being serially mounted to the lower end of the electric cylinder. This arrangement absorbs high-frequency road vibrations and reduces impact loads on the electric cylinder. Agricultural radial tyres [29] are selected to enhance traction and grip on muddy, soft terrain, meeting the grip requirements for mountainous, loose soils and preventing slippage during operation.

## 3. Suspension Model Construction

### 3.1. Kinematic Model

**Figure 4:**
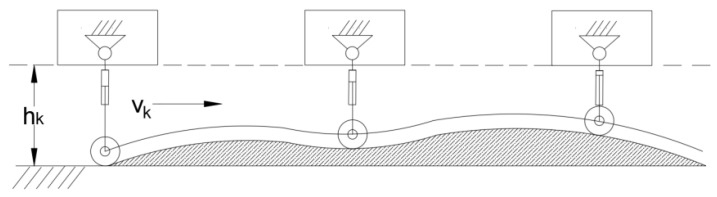
Illustration of maintaining chassis height.

To maintain a constant chassis height, the extension length of the electric cylinder rod must be adjusted, thereby altering the position of the tyres relative to the chassis. An analysis of the quarter suspension is now conducted to establish the relationship between the electric cylinder’s extension length and the chassis’ vertical displacement. The schematic illustrating how the chassis height is maintained constant through electric cylinder extension at a steady horizontal speed is shown in Figure 4. $h_{k}$: ground clearance; $V_{k}$: horizontal velocity.

**Figure 5:**
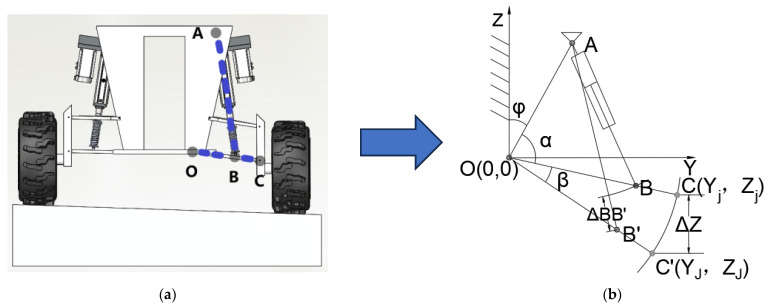
Schematic Diagram of Rear Right Levelling Suspension. (**a**) Physical prototype structure; (**b**) Kinematic analysis model.

The levelling suspension structure and connection relationships on the right side of the chassis are illustrated in Figure 5. A coordinate system is established with point O as the origin, OZ as the Z-axis, and OY as the Y-axis, as shown in Figure 5. Points O and A are connected to the chassis, with their coordinates known. The suspension parameters are detailed in Table 3. Here, AB represents the electric cylinder, spring, and damper assembly. When the system reaches equilibrium, changes in spring and damper length are neglected. OC denotes the lower control arm, which rotates about point O as the electric cylinder extends or retracts. Point O is the connection point between the lower control arm and the chassis; point B is the connection point between the damper and the lower control arm; Point C represents the connection between the lower control arm and the wheel. Its displacement along the Z-axis corresponds to the wheel’s relative displacement from the chassis. Therefore, this study employs the displacement of point C along the Z-axis as the variable representing the displacement change between the chassis and the electric cylinder’s support point A. As illustrated, ΔZ denotes the displacement of the rear right wheel along the Z-axis, i.e., the displacement at support point A. When point C rises (or falls) by ΔZ along the Z-axis, point A descends (or ascends) by ΔZ.

Given the displacement ΔZ of the rear right wheel along the Z-axis, the extension/retraction amount ΔBB′ of the electric cylinder must be controlled to maintain a constant chassis height. As illustrated in Figure 5, let α denote the angle between OA and the lower control arm, β denote the rotation angle of the lower control arm about point O, and φ denote the angle between OA and the Z-axis. The lengths of sides OA and OB in triangle OAB are known and constant. The length of side AB (or AB’) can be determined by combining the lengths of the electric cylinder and damper with the extension measured by the encoder.

Referring to Figure 5, in triangle OAB, by the cosine theorem:

(1) $$\cos\alpha =\frac{|\vec{OA}{|}^{2}+|\vec{OB}{|}^{2}-|\vec{AB}{|}^{2}}{2|\vec{OA}||\vec{OB}|}$$

From Equation (1) it follows that

(2) $$\alpha =\arccos\frac{|\vec{OA}{|}^{2}+|\vec{OB}{|}^{2}-|\vec{AB}{|}^{2}}{2|\vec{OA}||\vec{OB}|}$$

OC′ is obtained by rotating OC about point O, with points C and C′ taking coordinates (Y_j_, Z_j_) and (Y_J_, Z_J_) respectively, where

(3) $$Y_{j}=|\vec{\mathrm{OC}}|\cos(\alpha +\phi -\frac{\pi }{2})=|\vec{\mathrm{OC}}|\sin(\alpha +\phi )$$

(4) $$Z_{j}=|\vec{\mathrm{OC}}|\sin(\alpha +\phi -\frac{\pi }{2})=|\vec{\mathrm{OC}}|\cos(\alpha +\phi )$$

From the rotation matrix, we obtain

(5) $$\left(\begin{array}{c} Y_{J}\\ Z_{J} \end{array}\right)=\left[\begin{array}{cc} \cos\beta & -\sin\beta \\ \sin\beta & \cos\beta \end{array}\right]\left[\begin{array}{c} Y_{j}\\ Z_{j} \end{array}\right]=\left[\begin{array}{cc} \cos\beta & -\sin\beta \\ \sin\beta & \cos\beta \end{array}\right]\left[\begin{array}{c} \left|\vec{\mathrm{OC}}|\sin(\alpha +\phi \right)\\ \left|\vec{\mathrm{OC}}|\cos(\alpha +\phi \right) \end{array}\right]$$

The Z-coordinate of point C′ can be obtained by adding the required displacement ΔZ to the Z-coordinate of point C.

(6) $$Z_{J}=Z_{j}+\Delta Z=|\vec{\mathrm{OC}}|\cos(\alpha +\phi )+\Delta Z=|\vec{\mathrm{OC}}|\sin(\alpha +\phi )\sin\beta +|\vec{\mathrm{OC}}|\cos(\alpha +\phi )\cos\beta$$

The value of β can be determined as

(7) $$\beta =\alpha +\phi -\arccos\left(\cos(\alpha +\phi )+\frac{\Delta Z}{\left|\vec{\mathrm{OC}}\right|}\right)$$

From the lengths of sides OA and OB’ in triangle OAB′ and angles ∠A′ and ∠B′, the length of side AB′ may be determined by the cosine theorem. Thus, the length of the electric cylinder after extension is

(8) $$\left|\vec{AB\prime }\right|=\sqrt{|\vec{OA}{|}^{2}+|\vec{\mathrm{OB}\prime }{|}^{2}-2|\vec{OA}||\vec{\mathrm{OB}\prime }|\cos(\alpha +\beta )}$$

The stroke of the electric cylinder is

(9) $$\Delta \mathrm{BB}\prime =|\vec{AB\prime }|-|\vec{AB}|$$

### 3.2. Dynamic Model

The suspension is the mechanical assembly connecting the vehicle body to the wheels, primarily comprising springs, shock absorbers, active suspension actuators, and guidance mechanisms [30]. The suspension system of agricultural machinery buffers and absorbs body vibrations caused by uneven road surfaces while transmitting driving and braking forces between the wheels and the road. When operating conditions change, the suspension system also withstands inertial forces generated in all directions. A well-performing suspension system enables the vehicle to maintain optimal working conditions on complex terrain, ensuring driving smoothness and safety while sustaining ideal operating speeds [31].

Suspension systems are primarily categorised into three fundamental types: passive, active, and semi-active [32]. Passive suspension comprises springs that support the body and resist impacts, shock absorbers that dampen vibrations, and guidance mechanisms that restrict suspension movement. When appropriately parameterised, it effectively absorbs ground shocks. Active suspension builds upon passive suspension by incorporating actuators to provide additional energy input. Sensors mounted on the vehicle body continuously gather driving data, dynamically adjusting the energy input from the actuators according to varying road conditions. This ensures the suspension system adapts to different operational requirements. Semi-active suspension may be regarded as a system comprising variable-stiffness springs or variable-damping shock absorbers. It can dynamically adjust spring stiffness or damper damping in real time according to pre-programmed sequences within the vehicle’s onboard computer. Consequently, the suspension system can respond differently to road input in real time, thereby effectively controlling spring-mounted mass vibrations [33].

The type of suspension system directly influences the automatic levelling performance of agricultural machinery, with significant performance differences between various suspension configurations. Passive suspension offers simple structure and low cost but exhibits poor adaptability, struggling to meet real-time body posture control demands. Semi-active suspension cannot inject energy into the system, severely limiting its real-time body posture control capability. Active suspension systems can inject energy into the suspension, precisely responding to body posture changes and better meeting real-time control requirements, making them a superior choice for agricultural machinery automatic levelling needs.

Considering that agricultural machinery operating in hilly and mountainous terrains must cope with complex ground disturbance environments featuring concurrent low-frequency disturbances (10–40 Hz) and high-frequency disturbances (140–200 Hz) [22], current levelling suspension designs predominantly focus solely on achieving levelling functionality, with insufficient consideration given to shock absorption design [18,20,34,35,36]. To mitigate the impact of ground disturbances on the levelling system during agricultural operations, a shock-absorbing spring and damper are serially connected at the electric cylinder rod. Through the synergistic action of elastic buffering and damping energy dissipation, the transmission of ground disturbances is reduced, ensuring the control precision and stability of the levelling system. This approach adapts to the operational demands of agricultural machinery in complex terrain.

**Figure 6:**
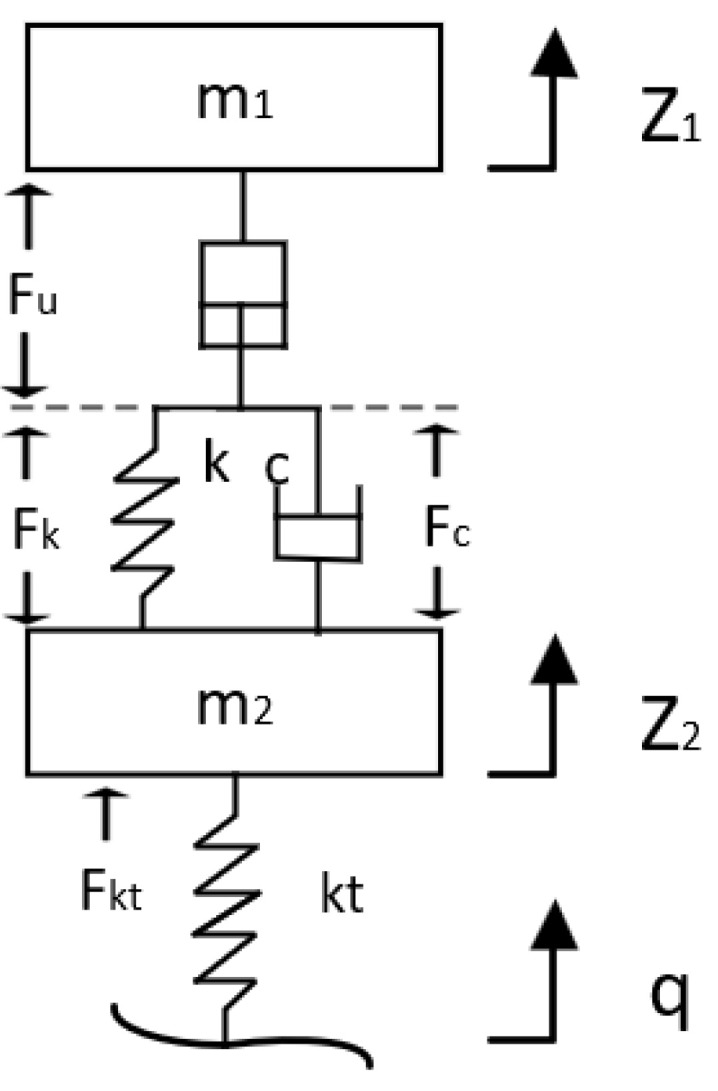
Quarter active suspension simplified model.

Modelling the suspension system is crucial for investigating active suspension control issues. Based on the aforementioned suspension geometric parameters, dynamic parameters such as spring-mounted and unsprung masses, spring force, and damping coefficient are incorporated to establish a two-degree-of-freedom dynamic model. This model analyses the suspension’s dynamic response under road excitation. A simplified quarter-wheel active suspension model is illustrated in Figure 6.

Based on the applied forces, the dynamic differential equations for the spring-mounted and spring-supported configurations may be, respectively, stated as:

(10) $$m_{1}{\ddot{z}}_{1}=F_{u}$$

(11) $$m_{2}{\ddot{z}}_{2}=-F_{k}-F_{c}+F_{\mathrm{kt}}$$

Among these:

(12) $$F_{u}=F_{k}+F_{c}$$

(13) $$F_{k}=k(Z_{2}-Z_{1}-S_{0})$$

(14) $$F_{c}=c(Z_{2}-Z_{1}-S_{0})$$

In the equation, m_1_ denotes the mass above the spring, m_2_ denotes the mass below the spring, Z_1_ denotes the displacement above the spring, Z_2_ denotes the displacement below the spring, k denotes the spring stiffness, c denotes the damper coefficient, Fu denotes the electric cylinder thrust, and S_0_ denotes the extension length of the electric cylinder rod.

Tyre dynamic models constitute a pivotal component in investigating the dynamic characteristics of suspension systems. Currently prevalent tyre models include point contact models, roller contact models, fixed footprint models, radial spring models, equivalent plane models, and annular models. The vertical forces, longitudinal forces, lateral forces, and steering moments acting upon the tyre play a crucial role in determining vehicle ride comfort, handling stability, and safety. Whilst more complex tyre models may better characterise tyre performance, the core objective of this study is to validate the vertical extension capabilities of the servo-electric cylinder-based active suspension system. The focus lies on the vertical dynamic response of the sprung mass, unsprung mass, and wheel hub under road excitation, rather than multi-directional force coupling issues such as longitudinal traction and lateral steering forces [37,38]. Other tyre models require additional integration of coupled longitudinal/lateral/vertical forces: on one hand, this leads to an exponential increase in the dimensionality of suspension dynamics equations, substantially amplifying computational redundancy; on the other, parameters unrelated to vertical levelling within complex models may obscure the core mechanism by which active suspension counteracts vertical terrain disturbances through electric cylinder adjustment, hindering clear validation of levelling performance. In contrast, the point contact model simplifies the tyre into a linear spring with vertical stiffness k_t_, offering a concise and intuitive representation that accurately characterises the tyre’s buffering effect against vertical road surface excitation. Furthermore, this model can be directly embedded into the two-degree-of-freedom dynamics equations without requiring additional iterative calculations. This simplifies model complexity while significantly enhancing simulation efficiency, enabling rapid output of key vertical levelling metrics for the active suspension. In summary, aligning with this study’s core objectives and model simplification rationale, forces acting on the tyre in directions other than the vertical plane are excluded from analysis. Consequently, the point contact model is employed to construct the suspension dynamics model. The mathematical expression for the point contact model is:

(15) $$F_{\mathrm{kt}}=k_{t}(q-Z_{2})$$

In the equation, k_t_ denotes tyre stiffness, q denotes the amplitude of road surface unevenness, and (q − Z_2_) denotes tyre compression.

Substituting the established suspension component model into the on-spring and off-spring dynamic differential equations yields the following expression:

(16) $$m_{1}{\ddot{z}}_{1}=k(Z_{2}-Z_{1}-S_{0})+c(Z_{2}-Z_{1}-S_{0})$$

(17) $$m_{1}{\ddot{z}}_{1}=-k(Z_{2}-Z_{1}-S_{0})-c(Z_{2}-Z_{1}-S_{0})+k_{t}(q-Z_{2})$$

## 4. Fuzzy PID Controller

### 4.1. Principles of Fuzzy Control

The traditional PID control algorithm is

(18) $$U(t)=K_{p}e(t)+K_{i}\int_{0}^{t}e(t)dt+K_{d}\frac{de(t)}{dt}$$

In the equation, U(t): Controller output; e(t): Controller input; $K_{p}$: Proportional gain; $K_{i}$: Integral gain; and $K_{d}$: Derivative gain.

Owing to the non-linearity, time-delay characteristics, and strong coupling inherent in agricultural machinery chassis levelling systems, conventional PID control algorithms employ fixed parameter values. Under such standard PID control, alterations in operational conditions induce significant errors within the system, compromising control efficacy. Excessive errors may destabilise machinery operations and potentially pose hazards. Therefore, improvements are made using fuzzy control. The fuzzy PID controller enables flexible non-linear control, allowing the system control parameters to be adaptively tuned, thereby enhancing control accuracy.

Agricultural machinery chassis levelling systems exhibit characteristics such as non-linearity, time-delay behaviour, and strong coupling. When employing conventional PID control, the system generates significant errors that compromise control effectiveness. Excessive errors may adversely affect the stability of agricultural machinery operations and even pose safety hazards. Fuzzy PID controllers enable flexible non-linear control, allowing adaptive tuning of system parameters to enhance control precision.

**Figure 7:**
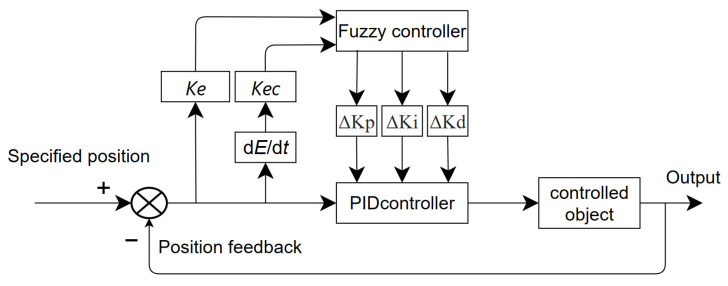
Fuzzy PID Control Principles.

The principle of fuzzy PID control is illustrated in Figure 7. This paper employs a two-input, three-output fuzzy controller, utilising both the chassis height deviation e and the deviation rate of change e_c_ as inputs. The input undergoes operations including fuzzyfying, fuzzy inference, and defuzzifying through the fuzzy controller, subsequently outputting the PID parameter control values $\Delta K_{p}$, $\Delta K_{i}$, and $\Delta K_{d}$. Based on the chassis driving state, these control parameters are adjusted in real time to maintain a constant chassis height, with the adjustment relationship detailed in Equation (19).

(19) $$\left\{\begin{array}{l} K_{p}=K_{p0}+\Delta K_{p}\\ K_{i}=K_{i0}+\Delta K_{i}\\ K_{d}=K_{d0}+\Delta K_{d} \end{array}\right.$$

In the formula: $K_{p0}$, $K_{i0}$, and $K_{d0}$ represent the initial values of the proportional coefficient, integral coefficient, and derivative coefficient, respectively, for the traditional PID controller.

### 4.2. Fuzzy PID Controller Design

During the initial design phase of the fuzzy PID controller, trial-and-error experiments were conducted to determine the optimal initial parameters for the PID controller. The final values established were: proportional coefficient (Kp) 50, integral coefficient (Ki) 6, and derivative coefficient (Kd) 4.

**Figure 8:**
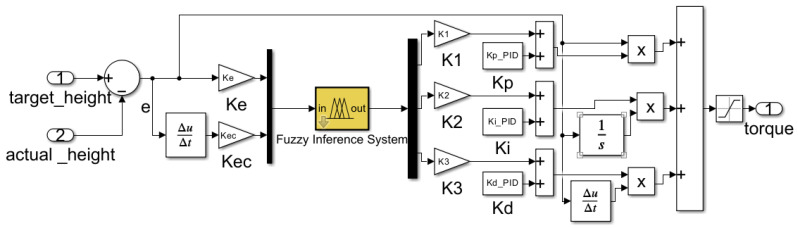
Fuzzy PID Controller Simulation Model.

**Figure 9:**
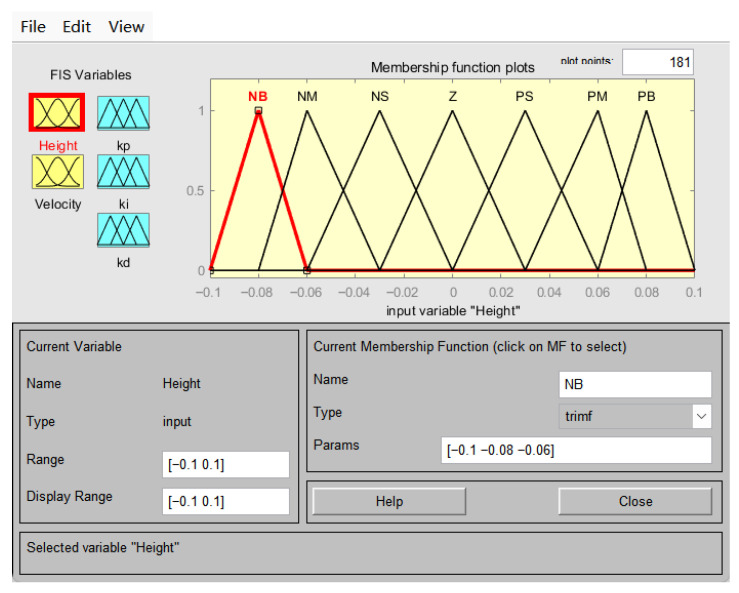
Input membership function.

The domain of the chassis height error (e) is set to [−0.1, 0.1], while the domain of the chassis height change rate (ec) is set to [−0.2, 0.2]. The domains of the output variables $\Delta K_{p}$, $\Delta K_{i}$, and $\Delta K_{d}$ are, respectively, set to [−6, 6], [−0.3, 0.3], and [−0.4, 0.4]. Subsequently, the continuous variables chassis height error (e), height change rate (ec), and output correction quantities ($\Delta K_{p}$, $\Delta K_{i}$, and $\Delta K_{d}$) were discretised into seven fuzzy linguistic variables: NB (negative large), NM (negative medium), NS (negative small), ZO (zero), PS (positive small), PM (positive medium), and PB (positive large). Based on the aforementioned parameter settings and fundamental principles of fuzzy control, a simulation model for the fuzzy PID controller was constructed, with its specific structure shown in Figure 8. The membership functions employed triangular functions, as illustrated in Figure 9.

The formulation of fuzzy rules centres on chassis height error (e) and chassis height change rate (ec) as core input variables. Based on the influence patterns of PID control parameters Kp, Ki, and Kd on the dynamic performance and steady-state accuracy of the control system, under varying e and ec input conditions, it is necessary to define the self-tuning criteria for output variables $\Delta K_{p}$, $\Delta K_{i}$, and $\Delta K_{d}$ [39,40,41] to ensure the controller dynamically optimises PID parameters based on chassis height deviation and trend, achieving precise control in complex operational scenarios.

(1) The magnitude of |e| indicates the distance of the chassis height from the setpoint. When |e| is large and positive, the chassis height deviation is significant. To rapidly reduce this deviation, response speed should be increased by setting $\Delta K_{p}$ to a large positive value and $\Delta K_{i}$ to a large positive value. When |e| is moderately positive, to sustain a relatively rapid reduction in deviation, $\Delta K_{p}$ should be set to a small positive value and $\Delta K_{i}$ to zero. When |e| is small, $\Delta K_{p}$ should be set to a small positive value and $\Delta K_{i}$ to a small negative value. When |e| is zero, the tilt angle is close to the preset value. To completely eliminate deviation and reach the set value while preventing system overshoot and vibration, $\Delta K_{p}$ should be set to a small negative value and $\Delta K_{i}$ to a large negative value.
(2) The magnitude of |ec| indicates the extent of non-uniform chassis height variation. When |ec| is large and positive, the chassis height may abruptly increase away from the preset value or rapidly decrease towards it. To suppress further deviation growth or prevent overshoot, $\Delta K_{d}$ is set to a large positive value. When |ec| is moderately positive, $\Delta K_{d}$ is set to a small positive value. When |ec| is zero, $\Delta K_{d}$ is set to a small negative value.
(3) When |e| is large and positive and |ec| is small and positive, to achieve rapid response and swift deviation reduction, $\Delta K_{p}$ is set to a small positive value, while $\Delta K_{i}$ and $\Delta K_{d}$ are set to zero.
(4) When |e| is small and positive while |ec| is large and positive, although |ec| is substantial, the chassis height remains near the setpoint range. To prevent overshoot, $\Delta K_{p}$ is set to a small negative value and $\Delta K_{i}$ to a small negative value. To suppress further increase in tilt angle, $\Delta K_{d}$ is set to a small positive value.

Based on the above fuzzy relationships, the fuzzy controller’s adjustment rules for variables $\Delta K_{p}$, $\Delta K_{i}$, and $\Delta K_{d}$ are derived, as shown in Table 4.

## 5. Control System Simulation Analysis

To validate the effectiveness of the suspension structure and control scheme, simulations must be conducted focusing on the chassis height control performance under random road surface excitation conditions. Road surface unevenness, typically used to describe the degree of undulation in the road surface, constitutes the primary excitation during vehicle operation. It not only directly determines the bumpiness level of road quality but also significantly impacts vehicle driving stability, ride comfort, and the service life of critical components [42]. Specifically, body vibrations induced by road surface irregularities cause tyre friction wear and increase dynamic suspension loads. This is particularly critical for vehicles operating in complex terrain, such as agricultural machinery in hilly or mountainous regions, where both chassis attitude control precision and operational efficiency are highly dependent on the ability to adapt to road surface irregularities. Numerous methods and instruments exist for measuring road surface irregularity. Classification by measurement reference includes fixed reference, follow-up reference, recursive reference, inertial reference, and angular reference; classification by measurement principle distinguishes between direct-contact measuring instruments and non-contact measuring instruments (responsive measuring instruments) [43]. Road surface irregularity measurement adheres to the ISO 8608 standard [44], which employs Power Spectral Density (PSD) as the core quantitative metric for road surface irregularity. This standard utilises Equation (20) to classify road surface grades.

(20) $$G_{q}\left(n\right)=G_{q}\left(n_{0}\right){\left(\frac{n}{n_{0}}\right)}^{-w}$$

In the equation, $G_{q}\left(n\right)$: denotes the power spectral density of the road; n: represents the spatial frequency of the road; W: denotes the frequency exponent; $n_{0}$: denotes the reference spatial frequency. Road surface roughness data obtained through standardised measurement must be converted into simulation-ready excitation signals via specialised modelling techniques. Commonly employed methods for modelling road surface roughness include: filtered white noise method, integrated white noise method, harmonic superposition method, inverse Fourier transform method, and wavelet analysis method [45].

**Figure 10:**
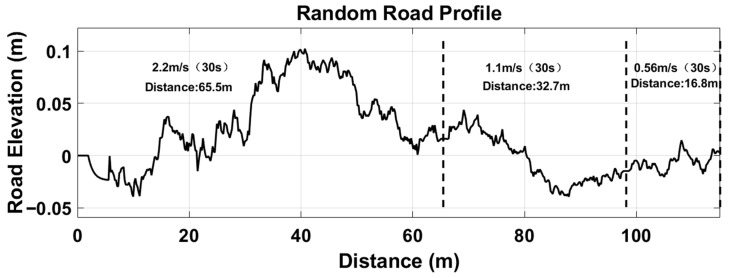
Random road surface time-domain excitation signal.

Given the complexity of hilly and mountainous terrains, fixed operating speeds struggle to reflect actual working conditions. Therefore, based on research into road surface unevenness characteristics and incorporating random road surface modelling methods, Matlab/Simulink was employed to generate random road surface time-domain excitation signals under segmented stable speeds. These segmented speeds were sequentially set at 2.2 m/s (8 km/h), 1.1 m/s (4 km/h), and 0.56 m/s (2 km/h). These speeds simulate typical operating conditions for agricultural machinery in hilly and mountainous regions, including high-speed, medium-speed, and low-speed operations. Figure 10 depicts the computed random road surface time-domain excitation signals, illustrating variations in road surface undulation across different segmented steady-state speeds. Such signals serve as excitation to simulate road surface conditions during agricultural machinery operations.

**Figure 11:**
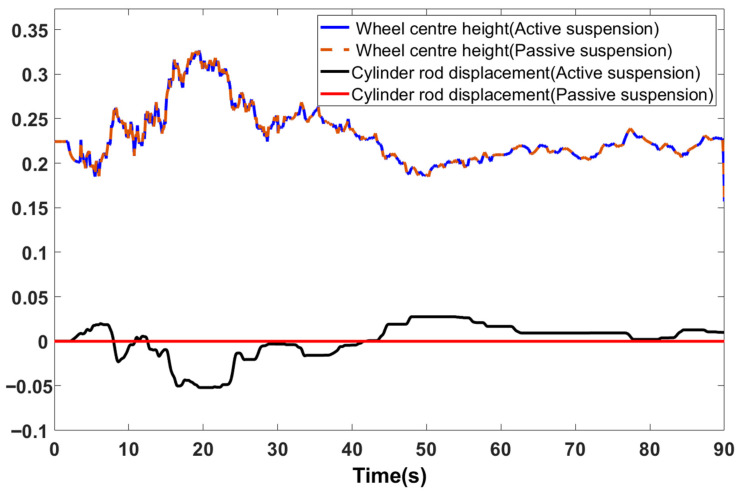
Comparison curve of electric cylinder rod displacement versus wheel centre height.

Matlab was employed to establish control system models for both passive suspension and active suspension based on fuzzy PID control, thereby clarifying the differences in chassis height control performance between the two suspension types. Passive suspension relies solely on passive damping through springs and damping elements. The active suspension control system sets the absolute chassis height relative to the initial position at h = 0.3 m. It employs fuzzy PID control to regulate the torque output of the servo electric cylinder, thereby maintaining a constant chassis height. During random road surface traversal, the comparison curve between wheel centre height and servo-electric cylinder rod displacement is shown in Figure 11. Both active and passive suspension systems exhibit wheel centre movement in response to road surface excitation. The passive suspension’s electric cylinder remains inactive, serving solely as a supporting link, whereas the active suspension automatically controls cylinder extension/retraction to adjust chassis height in response to excitation. The maximum wheel centre height of approximately 0.32 m corresponds to a 0.1 m road surface elevation, during which the electric cylinder rod retracts by 0.06 m. The minimum height of 0.18 m aligns with a 0.04 m road surface depression, where the rod extends by 0.03 m. This consistent height variation with road surface excitation confirms sustained effective contact between the wheel assembly and ground, eliminating any suspension lift. The displacement of the active suspension electric cylinder adjusts inversely to wheel centre displacement. When a road surface protrusion causes the wheel centre to rise, the cylinder rod retracts; when a road surface depression causes the wheel centre to lower, the cylinder rod extends. The cylinder rod displacement range of 0–0.1 m falls within the design stroke of 0–0.14 m, with no stroke exceeding limits. Furthermore, the phase difference between cylinder rod displacement adjustment and wheel centre displacement is ≤0.8 s, indicating rapid response speed.

**Figure 12:**
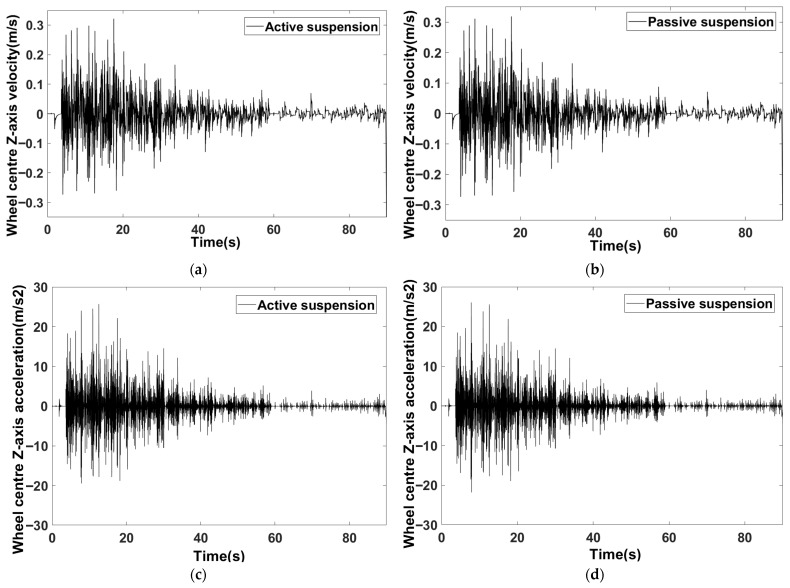
Wheel centre output parameters. (**a**) Wheel centre Z-axis velocity (active suspension). (**b**) Wheel centre Z-axis velocity (passive suspension). (**c**) Wheel centre Z-axis acceleration (active suspension). (**d**) Wheel centre Z-axis acceleration (passive suspension).

As shown in Figure 12, both active and passive suspension wheels maintain close contact with the road surface during travel, resulting in minimal differences in their velocity and acceleration curves. Examining the overall trend of the curves: 0–30 s: Corresponding to the high-speed segment at 2.2 m/s, wheel velocity and acceleration exhibit the greatest fluctuations, with velocity peaks approaching ±0.3 m/s and acceleration peaks nearing ±20 m/s^2^. 30–60 s: corresponding to the 1.1 m/s medium-speed segment, where fluctuations narrowed with peak speeds approaching ±0.1 m/s and peak accelerations nearing ±10 m/s^2^. 60–90 s: corresponding to the 0.6 m/s low-speed segment, where fluctuations further levelled off with peak speeds approaching ±0.05 m/s and peak accelerations nearing ±1 m/s^2^. Analysis of the wheel hub’s Z-axis velocity and acceleration curves reveals that the fluctuation characteristics align with the segmented speed variation logic: higher speeds induce more pronounced dynamic responses to road surface undulations, resulting in greater amplitude fluctuations in both velocity and acceleration. Furthermore, throughout the simulation, the wheel centre’s Z-axis velocity remained within ±0.3 m/s and Z-axis acceleration within ±20 m/s^2^, with smooth velocity curves exhibiting no abrupt changes. This confirms that the suspension springs and dampers effectively suppressed high-frequency road impacts.

**Figure 13:**
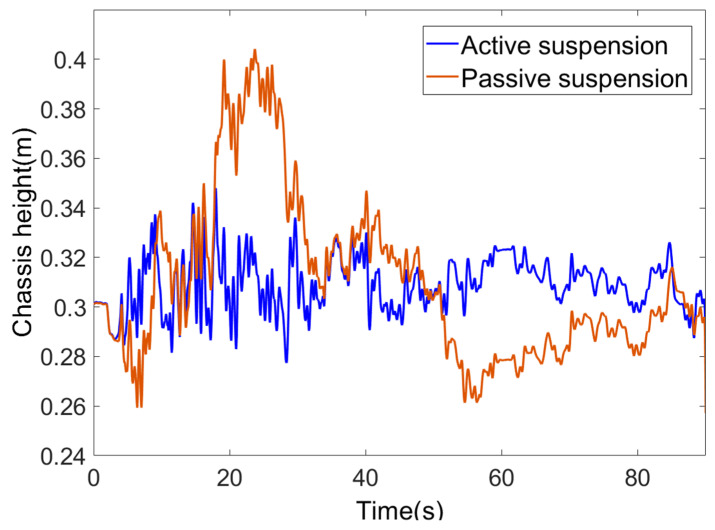
Variation in chassis height.

Changes in chassis height are illustrated in Figure 13. When combined with random road surface excitation at segmented stable speeds, chassis height fluctuations showed a positive correlation with speed: during the initial 30 s high-speed segment at 2 m/s, where road disturbances were most pronounced, passive suspension exhibited height variations ranging from 0.26 to 0.4 m. The maximum deviation from the target height of 0.3 m was approximately 0.1 m, whereas active suspension maintained deviations within ±0.05 m. As speed decreased to 1.2 m/s and 0.6 m/s, road disturbances diminished. The amplitude of height fluctuations in the passive suspension gradually narrowed but remained significantly greater than that of the active suspension. Under random road surface excitation, the passive suspension exhibits pronounced fluctuation characteristics. Calculations reveal an average absolute error of 0.025 m, a root mean square error of 0.035 m, and a peak error of 0.104 m, with a maximum deviation of approximately 0.1 m from the target height of 0.3 m. From a control perspective, passive suspension relies solely on passive damping via springs and dampers, lacking active chassis height regulation. Consequently, its height response is entirely governed by road surface excitation, exhibiting pronounced height deviations during both low-frequency depression sections (0–15 m and 80–110 m) and high-frequency elevation sections (30–50 m). The active suspension chassis height consistently oscillated around the target value of 0.3 m with minimal fluctuation, achieving an average absolute error of 0.011 m, a root mean square error of 0.014 m, and a peak error of 0.048 m. The maximum deviation was controlled within ±0.05 m. Evidently, under active suspension, the mean absolute error, root mean square error, and peak error all decrease, with chassis height stability significantly enhanced compared to passive suspension.

**Figure 14:**
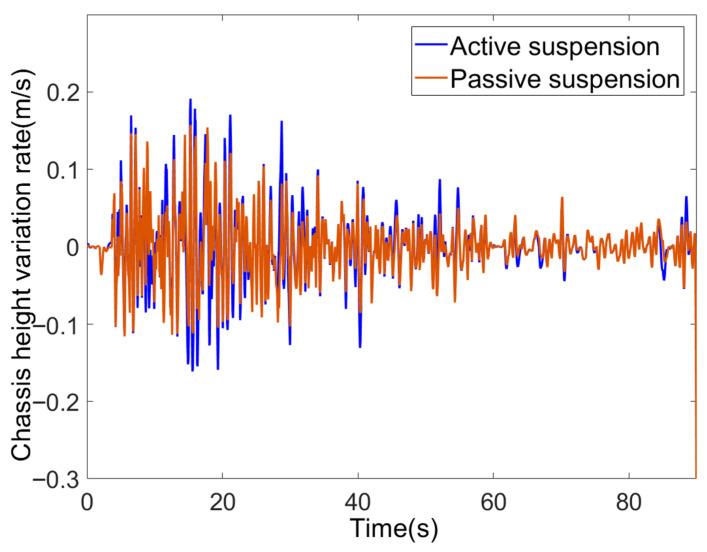
Chassis height variation rate.

The chassis height variation rate is illustrated in Figure 14. Fluctuations in the variation rate exhibit a positive correlation with vehicle speed: during the initial 30 s at 2 m/s, the most severe road disturbances occurred, with both active and passive suspension systems registering variation rate fluctuations of approximately ±0.1 m/s. As speed decreased to 1.2 m/s and 0.6 m/s, the fluctuation range of the variation rate progressively narrowed. Both variation rates were controlled within ±0.1 m/s without high-frequency oscillations, demonstrating the fundamental damping effect of springs and dampers in effectively filtering out the high-frequency components of road surface disturbances. Comparing the suspensions reveals that the active suspension exhibits a more dynamic response in its height variation rate curve: during the 0–20 s interval (corresponding to low-frequency potholes at high speeds) and the 30–40 s interval (high-frequency bumps), the active suspension’s rate fluctuations significantly exceed those of the passive suspension. This is due to the active suspension’s ability to dynamically adjust height via its electric cylinder, enabling proactive adaptation to road surface changes and enhancing resistance to complex disturbances. Passive suspension, relying solely on spring damping for passive buffering, exhibits relatively smoother rate-of-change fluctuations and weaker active adaptability to terrain variations. While both maintain relatively stable rate-of-change profiles due to spring and damper damping effects, the active suspension demonstrates greater rate-of-change responsiveness during intense road disturbances through its electric cylinder-driven adjustments, highlighting its advantage in actively countering terrain changes.

**Figure 15:**
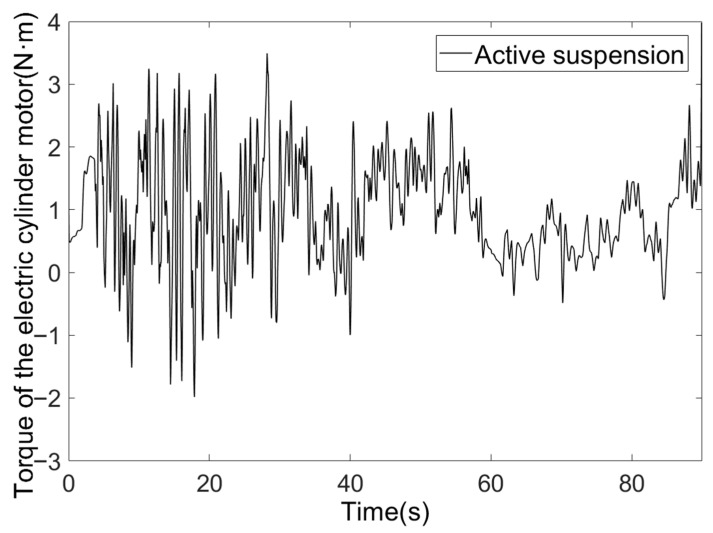
Torque of the electric cylinder motor.

**Figure 16:**
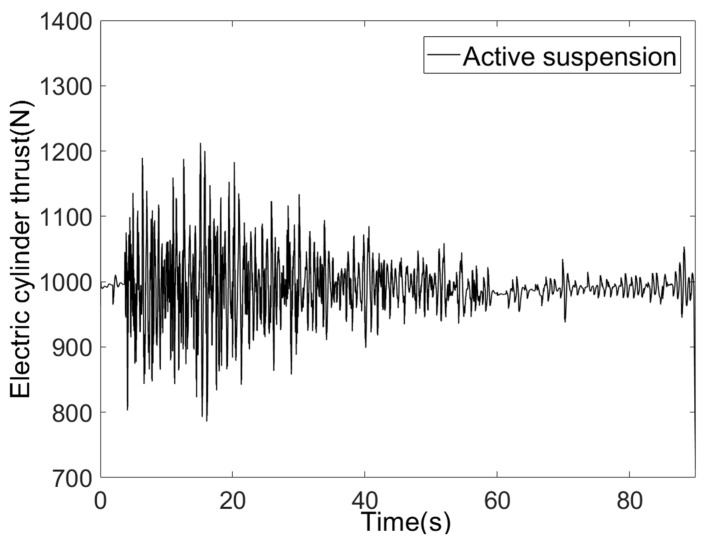
Electric cylinder thrust.

The torque output and thrust of the active suspension electric cylinder motor are shown in Figure 15 and Figure 16.

The electric cylinder motor’s output torque primarily falls within the ±3 N·m range, conforming to the motor’s rated torque of 4.8 N·m and instantaneous maximum torque of 14.4 N·m. Torque overshoot is absent, and the motor operates stably. The electric cylinder’s thrust dynamically varies with road surface excitation, primarily ranging between 0.9 and 1.1 KN. This remains below the rated thrust of 6.78 KN, eliminating overload risk, with the thrust curve synchronising with road surface excitation.

In summary, under random road surface excitation, the suspension active levelling system based on fuzzy PID control achieves active stabilisation of chassis height through real-time adjustment by servo electric cylinders. With random road surface undulations ranging approximately 0.15 m, the active suspension system strictly confined chassis height deviation within ±0.05 m. This represents a significant improvement over the ±0.1 m tolerance of passive suspension systems. Furthermore, the control system demonstrated a response time ≤ 0.8 s, exhibiting rapid responsiveness. This provides valuable reference for chassis levelling and vibration suppression in small agricultural machinery operating in hilly and mountainous terrains.

## 6. Discussion

Firstly, the servo electric cylinder-spring-shock absorber series suspension active levelling system designed in this study demonstrates certain advantages in the levelling performance of agricultural machinery on hilly terrain. It is now compared with two typical studies:

(1) Comparison with conventional hydraulic levelling systems: The transmission efficiency of the servo electric cylinder employed herein (≥90%) is nearly doubled compared to hydraulic cylinders (≤50%), thereby avoiding issues such as pipeline leakage in hydraulic systems, levelling lag caused by temperature-dependent oil viscosity, and suboptimal levelling precision. The system response time is ≤0.8 s, whereas hydraulic systems typically exhibit response times of 1.2–1.5 s, thereby enhancing overall system responsiveness. Moreover, electric cylinders dispense with complex components such as hydraulic pumps and reservoirs, offering reduced volume and mass compared to hydraulic systems, thereby better meeting the lightweight requirements of compact agricultural machinery.
(2) Comparison with semi-active levelling or passive adaptation: Systems such as parallelogram-type semi-active levelling driven by hydraulic cylinders with passive contouring, or passive adaptation via bending and twisting mechanisms, cannot accommodate compound slopes. This study demonstrates that servo electric cylinders employing fuzzy PID control can regulate output parameters in response to road surface excitation, providing real-time adaptation to terrain variations. This capability enables adaptation to diverse operational conditions, offering valuable reference for omnidirectional levelling under compound slopes (roll + pitch).

Secondly, to validate the core mechanism of vertical suspension levelling, this paper employs a simplified two-degree-of-freedom quarter-suspension model coupled with a point-contact tyre model. While this currently suffices for preliminary vertical dynamics analysis, the model exhibits significant limitations in complex hilly terrain operations:

(1) The simplified tyre model neglects lateral dynamic coupling effects. The point-contact model accounts solely for vertical tyre stiffness and compression, disregarding lateral deflection forces and restoring moments under cross-slope conditions. During hilly terrain operations, substantial lateral forces induce chassis roll, causing single-side wheel deflection forces. Consequently, measured vertical suspension displacements deviate from actual road surface excitation, leading to compensation errors in the electric cylinder levelling system.
(2) The quarter-suspension model disregards multi-wheel coupling effects. This study establishes dynamic equations solely for single-wheel suspension, neglecting load transfer across all four wheels during complex terrain conditions. For instance, when front wheels traverse pits, body pitching induces additional loads on rear suspension. In practical agricultural machinery operation, uneven multi-wheel load distribution causes electric cylinder thrust overshoot, potentially leading to structural damage during prolonged operation. The current model fails to simulate such multi-wheel coordination issues.
(3) The road surface excitation model excludes unstructured terrain features. This paper generates random road surface excitation based on the ISO 8608 standard, omitting irregular disturbances common in hilly terrain such as protruding rocks, potholes, and subsidence in soft soil. Soft soil causes variations in tyre contact area.

Finally, based on the aforementioned limitations and research findings, investigations and improvements will be pursued across multiple fronts:

(1) Development of a multi-degree-of-freedom vehicle model: The quarter-suspension model will be expanded into a seven-degree-of-freedom vehicle model, incorporating three global degrees of freedom (vertical, roll, and pitch) and four independent vertical degrees of freedom for each wheel. Coupled dynamic equations will be derived from Lagrange’s equations to clarify the coupling relationships between pitch and roll. The Pacejka tyre model will be incorporated, inputting parameters such as camber angle, vertical load, and tyre pressure to output lateral forces, steering moments, and longitudinal adhesion. This addresses the limitations of the current point-contact model.
(2) Multi-wheel Cooperative Control: The control algorithm employs a master-slave configuration. The master controller acquires the vehicle’s overall roll and pitch angles via dual-axis inclinometers. Integrating this with the full vehicle dynamics model, it calculates the coupled disturbance compensation required for each wheel. Concurrently, it dynamically adjusts weighting factors based on vertical load feedback from each wheel, preventing single-wheel overload. Each wheel suspension employs an independent slave controller executing fuzzy PID control. This tracks local target height while receiving coupled compensation values from the master controller, enabling real-time adjustment of electric cylinder extension/retraction.

## 7. Conclusions

(1) An active levelling system for small agricultural machinery chassis in hilly terrain was designed based on a fuzzy PID algorithm, proposing a ‘servo electric cylinder-spring-shock absorber series configuration’ solution. Kinematic and dynamic models were established based on the installation position and operational state of the electric cylinder within the quarter-link suspension. The kinematic model yielded the relationship between the vertical displacement of the electric cylinder’s support point and the extension length of its rod.
(2) A comparative simulation experiment was conducted between a passive suspension and an active suspension. The simulation results demonstrated that the active levelling system based on fuzzy PID control strictly confined the actual chassis height deviation to within ±0.05 m when traversing random road surfaces, representing a significant improvement in chassis stability compared to the passive suspension. The chassis height variation rate remained within ±0.2 m/s, demonstrating the shock absorption capabilities of the spring and damping components. The electric cylinder response time was ≤0.8 s, exhibiting rapid responsiveness.
(3) This research provides agricultural machinery manufacturers with a lightweight, highly efficient, and low-complexity active levelling solution. Its simplified structural design and precise control logic can facilitate the development of compact agricultural machinery for hilly and mountainous terrains, thereby advancing the mechanisation of agriculture in such regions.

## Figures and Tables

**Table 1:** Comparison of Agricultural Machinery Levelling Techniques for Hilly and Mountainous Terrains.

Levelling Method	Levelling Direction	Active/Passive Levelling	Structural Complexity	Terrain Adaptability
Hydraulic differential height type	Lateral only (roll)	Active (hydraulic cylinder driven)	Requires connecting rods, hydraulic pumps, multiple sets of hydraulic cylinders, control valve assemblies, hydraulic piping and reservoirs, occupying substantial space and featuring a complex structure.	Suitable only for single gentle lateral slopes; not applicable to longitudinal slopes or compound slopes (roll + pitch)
Parallel four-bar linkage	Lateral only (roll)	Semi-active (hydraulic cylinder driven + passive terrain adaptation)	Requires a parallel four-bar linkage mechanism, multiple sets of hydraulic cylinders, control valve assemblies, hydraulic piping and reservoirs, occupying substantial space and featuring a complex structure.	Suitable only for single gentle horizontal slopes; not applicable to longitudinal slopes or compound slopes
Adjustable centre of gravity mechanism	Lateral (roll) + Longitudinal (pitch)	Semi-active (motor-driven centre of gravity shift + passive terrain adaptation)	Requires both horizontal and vertical guide rails, drive motors; simple structure, but occupies considerable space.	Suitable for composite slope (roll + pitch)
Bending and twisting the waist	No active levelling direction, only passive adaptation to terrain undulations	Passive (propelled by terrain forces acting upon the articulated shaft/twisting of the waist shaft)	The design of a central waist hinge shaft, a waist-contouring shaft, and their associated structures is required, presenting a complex construction.	Suitable for rough terrain, but unable to handle steep slopes or compound slopes (roll + pitch)
Omnidirectional levelling	Lateral (roll) + Longitudinal (pitch)	Semi-active (motor-driven centre-of-gravity shift + passive contouring)	multiple sets of hydraulic cylinders, control valve assemblies, hydraulic piping and reservoirs, occupying substantial space and featuring a complex structure.	Suitable for compound slopes (roll + pitch) and uneven terrain

**Table 2:** Differences Between Hydraulic Cylinders and Electric Cylinders.

	Hydraulic Cylinders	Electric Cylinder
Transmission medium	Hydraulic oil	Mechanical structure
Transmission efficiency	≤50%	≥90%
Operating temperature	The operating temperature range for hydraulic cylinders is typically specified as −40 to 120 °C, with performance being susceptible to fluctuations in temperature.	The operating temperature range for electric cylinders is typically specified as −30 to 80 °C, with minimal impact on performance from temperature fluctuations.
Structural complexity	Requires hydraulic pumps, hydraulic cylinders, control valve assemblies, hydraulic piping and reservoirs, occupying considerable space and featuring complex structures.	Requires electric motors and mechanical transmission components, occupies minimal space, facilitates convenient layout, and features a simple structure.
Position controllability	difficulties	easily
Maintenance workload	Large	small
Environmental pollution	Hydraulic oil leakage	small

**Table 3:** Suspension parameters.

Name	Value (m)
Y_A_	0.1
Z_A_	0.6
OA	0.608
OB	0.18
BC	0.12

**Table 4:** Fuzzy control rules.

	e_c_	ΔK_p_/ΔK_i_/ΔK_d_						
e		NB	NM	NS	ZO	PS	PM	PB
NB		PB/NB/PS	PB/NB/NS	PM/NM/NB	PM/NM/NB	PS/NS/NB	ZO/ZO/NM	ZO/ZO/PS
NM		PB/NB/PS	PB/NB/NS	PM/NM/NB	PS/NS/NM	PS/NS/NM	ZO/ZO/NS	NS/ZO/ZO
NS		PM/NB/ZO	PM/NM/NS	PM/NS/NM	PS/NS/NM	ZO/ZO/NS	NS/PS/NS	NS/PS/ZO
ZO		PM/NM/ZO	PM/NM/NS	PS/NS/NS	ZO/ZO/NS	NS/PS/NS	NM/PM/NS	NM/PM/ZO
PS		PS/NM/ZO	PS/NS/ZO	ZO/ZO/ZO	NS/PS/ZO	NS/PS/ZO	NM/PM/ZO	NB/PB/ZO
PM		PS/ZO/PB	ZO/ZO/NS	NS/PS/PS	NM/PS/PS	NM/PM/PS	NM/PB/PS	NB/PB/PB
PB		ZO/ZO/PB	ZO/ZO/PM	NM/PS/PM	NM/PM/PM	NM/PM/PS	NB/PB/PS	NB/PB/PB

## Data Availability

The original contributions presented in the study are included in the article. Further inquiries can be directed to the corresponding author.

## Abbreviations

The following abbreviations are used in this manuscript:

MCU	Microcontroller Unit
